# Prognostic Value of Classifying Parapharyngeal Extension in Nasopharyngeal Carcinoma Based on Magnetic Resonance Imaging

**DOI:** 10.1155/2015/749515

**Published:** 2015-03-26

**Authors:** Guo-Yi Zhang, Ying Huang, Xue-Feng Hu, Xiang-Ping Chen, Tao Xu, Li-Zhi Liu, Wei-Hong Wei, Guo-Sen Huang, Miao-Miao Zhou, Ze-Li Huang, Yue-Jian Wang

**Affiliations:** ^1^Cancer Center, Cancer Research Institute, Foshan Hospital, Sun Yat-sen University, Foshan, Guangdong 528000, China; ^2^Department of Radiation Oncology, State Key Laboratory of Oncology in Southern China, Collaborative Innovation Center of Cancer Medicine, Cancer Center, Sun Yat-sen University, Guangzhou, Guangdong 510060, China; ^3^Imaging Diagnosis and Interventional Center, State Key Laboratory of Oncology in Southern China, Collaborative Innovation Center of Cancer Medicine, Cancer Center, Sun Yat-sen University, Guangzhou, Guangdong 510060, China

## Abstract

*Purpose*. To subclassify parapharyngeal extension in nasopharyngeal carcinoma (NPC) and investigate its prognostic value and staging categories based on magnetic resonance imaging (MRI). *Methods and Materials*. Data from 1504 consecutive NPC patients treated with definitive-intent radiotherapy were analyzed retrospectively. Sites of parapharyngeal extension were defined by MRI. Overall survival (OS), local relapse-free survival (LRFS), and distant metastasis-free survival (DMFS) were calculated by the Kaplan-Meier method and compared with the log-rank test. Hazard consistency and hazard discrimination were determined by multivariate analysis with Cox proportional hazards models. *Results*. 1104 patients (73.4%) had parapharyngeal extension; 1.7–63.8% had involvement of various anatomic sites. The hazard ratio for death was significantly higher with extensive parapharyngeal extension (lateral pterygoid muscle of masticator space and beyond or parotid space) than with mild extension (medial pterygoid muscle of masticator space, or carotid, prestyloid, and prevertebral or retropharyngeal space). OS, LRFS, and DMFS with extensive parapharyngeal extension were similar to those in T4 disease; OS, LRFS, and DMFS with mild parapharyngeal extension were significantly higher than in those T3 disease (all *P* ≤ 0.015). *Conclusions*. Parapharyngeal extension in NPC should be subclassified as mild or extensive, which should be regarded as stages T2 and T4 diseases, respectively.

## 1. Introduction

**Figure 1:**
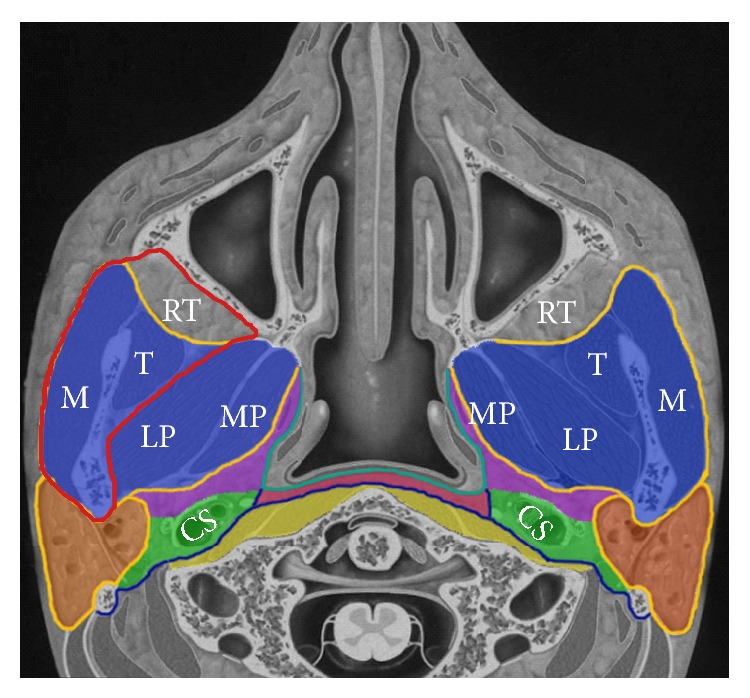
Axial section through the nasopharynx showing the relationship between various anatomic sites of parapharyngeal extension surrounding the nasopharynx, which are divided by the three layers of the cervical fascia: deep (dark blue line), middle (bright green line), and superficial (orange line). Parapharyngeal extension includes involvement of the masticator space (MS, blue), prestyloid space (PSS, purple), carotid space (CS, green), retropharyngeal space (RPS, pink), prevertebral space (PVS, yellow), and parotid space (PS, brown). Compared with the anatomic masticator space, the staging masticator space (the region within the red line) excludes the medial and lateral pterygoid muscles but includes the retromaxillary fat pad. MP: medial pterygoid muscle; LP: lateral pterygoid muscle; M: masseter muscle; T: temporalis muscle; RT: retromaxillary fat pad.

Nasopharyngeal carcinoma (NPC) has a strong invasive tendency and often develops parapharyngeal extension, with a prevalence of 72–83% at diagnosis [1–10]. Parapharyngeal extension in NPC indicates tumor invasion beyond the pharyngobasilar fascia and into the parapharyngeal, masticator, prevertebral, or parotid space, which are separated by the cervical fascias; the parapharyngeal space can be subdivided into the prestyloid, carotid, and retropharyngeal spaces (Figure 1).

The prognostic significance of parapharyngeal extension in NPC has not been resolved [1–12] and the recommendations in published staging systems pertaining to parapharyngeal extension are ambiguous [13–16]. Chua et al. [1] reported that lateral tumor invasion beyond the line joining the free edge of the medial pterygoid plate to the styloid process was an independent prognostic factor. Other studies have shown that paraoropharyngeal extension or posterolateral tumor invasion beyond a line drawn from the styloid process to the midpoint of the posterior edge of the great occipital foramen also has some value as an indicator of poorer prognosis [3–5]. However, using the American Joint Committee on Cancer (AJCC) definition of parapharyngeal extension beyond the pharyngobasilar fascia or subclassifying the degree of parapharyngeal extension according to the presence or absence of carotid space involvement, parapharyngeal extension had no prognostic significance in NPC [7–10]. The definitions of parapharyngeal extension used in these studies vary widely, and most patients were staged on the basis of computed tomography (CT) scans. Conversely, two studies based on magnetic resonance imaging (MRI) suggested that involvement of any part of the anatomic masticator space should be regarded as T4 disease [11, 12].

In light of these discrepancies, this study aimed to identify appropriate subclassifications (lateral, posterior, posterolateral, and inferior spread) for parapharyngeal extension in NPC and to reassess the prognostic value and staging categories for parapharyngeal extension based on MRI in patients with NPC treated with definitive-intent radiotherapy.

## 2. Materials and Methods

### 2.1. Patients

This retrospective study was approved by the institutional review board and all examinations were performed after written informed consent had been obtained from the patients or their next of kin. From December 2002 to October 2006, 1504 ethnic Chinese patients (1145 males, mean age 46 years, range 13–76 years; 358 females, mean age 43 years, range 18–75 years) with newly diagnosed, untreated, and nondisseminated NPC who were subsequently treated with definitive-intent radiotherapy were enrolled in the study. Patients whose treatment deviated from institutional guidelines due to advanced age or organ dysfunction were excluded. All patients underwent pretreatment evaluation including MRI of the neck and nasopharynx, chest radiography, abdominal sonography, and a whole-body bone scan. Medical records and imaging studies were reviewed and all patients were restaged according to the 2010 AJCC TNM staging system for NPC [15].  Table 1 shows the patients' pathologic classification, T classification, N classification, and overall stage.

### 2.2. Imaging Protocol

All patients underwent MRI using a 1.5 T system (Signa CV/I; GE Healthcare, Milwaukee, WI) employing a spin echo technique. The region from the suprasellar cistern to the inferior margin of the sternal end of the clavicle was examined with a head and neck combined coil. T1-weighted images in the axial, coronal, and sagittal planes (repetition time 500–600 ms, echo time 10–20 ms) and T2-weighted images in the axial plane (repetition time 4000–6000 ms, echo time 95–110 ms) were obtained before injection of the contrast material. After intravenous injection of Gd-DTPA at a dose of 0.1 mmol/kg body weight, T1-weighted axial and sagittal sequences and T1-weighted fat-suppressed coronal sequences were performed sequentially, with parameters similar to those used before Gd-DTPA injection. The section thicknesses and interslice gaps were 5 mm and 1 mm for the axial plane and 6 mm and 1 mm for the coronal and sagittal planes.

### 2.3. Image Assessment

Two experienced radiologists (Y.Z.L. and L.Z.L., with 10 and 12 years' experience in MRI of NPC, resp., at the time of the study) separately evaluated the images. Any disagreements were resolved by consensus. MRI findings of parapharyngeal extension were assessed at the following sites: medial pterygoid muscle, lateral pterygoid muscle, staging masticator space (tumor involvement beyond the anterior surface of the lateral pterygoid muscle), prestyloid space, retropharyngeal space, anterior carotid space, posterior carotid space (the demarcation of the anterior and posterior carotid space was the posterior edge of the internal jugular vein), prevertebral space, parotid space, and paraoropharyngeal space (Figure 1). Paraoropharyngeal extension was defined as parapharyngeal involvement of tumor below the C1/C2 interspace [3].

### 2.4. Treatment

All patients were treated with definitive-intent radiotherapy. Most of the patients (805, 53.5%) were treated with conventional techniques based on CT simulation, but 621 (41.3%) patients received intensity-modulated radiation therapy (IMRT) and 78 (5.2%) underwent three-dimensional conformal radiotherapy (3DCRT). Details of the radiotherapy techniques used at our cancer center have been reported previously [17–19].

During the study period, based on the AJCC 2002 staging system, our institutional guidelines recommended radiotherapy alone for stage I disease, concomitant chemoradiotherapy (CCRT) for stage II disease, and induction or adjuvant chemotherapy together with CCRT for stage III to IVB disease. Overall, 101 study patients were treated with radiotherapy only and 317 received CCRT; induction chemotherapy and CCRT were delivered to 423 patients and adjuvant chemotherapy and CCRT were delivered to 663 patients.

### 2.5. Followup and Statistical Analysis

Complete follow-up data were available for 97.4%, 96.5%, and 73.6% of the 1504 patients at 3 years, 5 years, and 8 years, respectively. The duration of followup was calculated from the first day of treatment to either the day of death or the day of the last examination. Patients were followedup at least once every 3 months during the first 2 years and every 6 months thereafter until death. The median follow-up period was 110 months (range 2–128 months).

SPSS version 16.0 (IBM, Armonk, NY) was used for all statistical analyses and the timing of all events was measured from the start of treatment. The time to the first defining event was assessed for the following end points: overall survival (OS), local relapse-free survival (LRFS), and distant metastasis-free survival (DMFS). Actuarial rates were calculated by the Kaplan-Meier method and compared using the log-rank test [20]. Multivariate analyses using Cox proportional hazards models were employed to test hazard consistency and hazard discrimination by backward elimination of insignificant explanatory variables [21]. Two-tailed *P* values of <0.05 were considered statistically significant.

## 3. Results

### 3.1. Pattern of Failure

One hundred and seventy patients (11.3%) developed local recurrence, 220 (14.6%) developed distant metastases, and 446 (29.7%) died. For the entire patient population, 5-year OS, LRFS, and DMFS ± standard deviation were 80.9% ± 1.0%, 89.7% ± 0.8%, and 86.3% ± 0.9%, respectively, and 8-year OS, LRFS, and DMFS were 70.3% ± 1.2%, 87.6% ± 0.9%, and 84.4% ± 1.0%, respectively.

### 3.2. Prevalence of Parapharyngeal Extension by Anatomic Site

Of the 1504 patients, 1104 (73.4%) were diagnosed with parapharyngeal extension. Among these 1104 patients, the retropharyngeal and prestyloid spaces were the most commonly involved sites, followed by the prevertebral space, anterior carotid space, medial pterygoid muscle, lateral pterygoid muscle, posterior carotid space, paraoropharyngeal space, parotid space, and staging masticator space, with incidences of 63.8% (960/1504), 62.5% (940/1504), 39.0% (587/1504), 29.4% (442/1504), 20.7% (311/1504), 8.8% (132/1504), 7.4% (111/1504), 3.3% (50/1504), 2.6% (39/1504), and 1.7% (26/1504), respectively. The most common sites of tumor involvement were thus adjacent to the nasopharynx and the least common were more distant to the nasopharynx.

### 3.3. Subclassification of Masticator, Carotid, and Paraoropharyngeal Space Extension

The prognostic value of the grade of lateral tumor spread was analyzed to determine an optimal subclassification for masticator space invasion. There was no significant difference in hazard ratio (HR) for OS, LRFS, or DMFS between patients with lateral pterygoid muscle invasion and those with staging masticator space invasion (*P* = 0.727, *P* = 0.765, and *P* = 0.842, resp.), whereas patients with medial pterygoid muscle invasion had significantly lower HRs for death and distant metastasis than those with lateral pterygoid muscle invasion (*P* = 0.006 and *P* = 0.011, resp.) (Table 2). When masticator space invasion was graded as involvement of the medial pterygoid muscle versus involvement of the lateral pterygoid muscle and beyond, significant differences in HRs for death and distant metastasis were observed between the two groups (*P* = 0.005 and *P* = 0.006, resp.; Table 2).

To investigate the prognostic significance of posterolateral or inferior tumor spread, we compared differences in HR between anterior and posterior carotid space involvement and between paranasopharyngeal and paraoropharyngeal extension. The HRs for OS, LRFS, and DMFS in patients with anterior carotid space invasion were similar to those of patients with posterior carotid space invasion (*P* = 0.618, *P* = 0.621, and *P* = 0.085, resp.), as were these values for patients with paraoropharyngeal extension compared with patients with paranasopharyngeal extension (*P* = 0.482, *P* = 0.072, and *P* = 0.312, resp.) (Table 2). These results suggest that subclassifications of carotid space or paraoropharyngeal extension are unnecessary.

### 3.4. Classification of Parapharyngeal Extension

**Figure 2:**
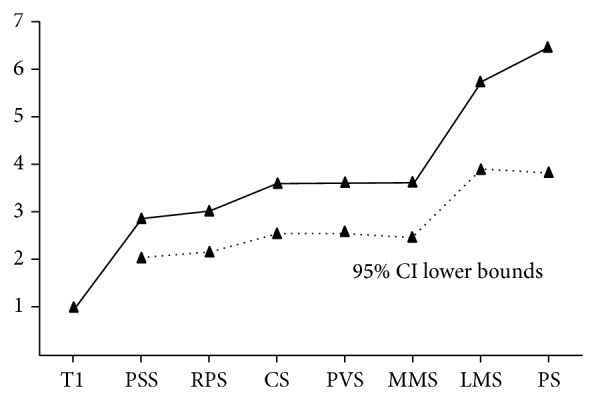
HR for death according to anatomic site of parapharyngeal extension (HR adjusted for age, gender, chemotherapy, and N category according to the seventh edition AJCC system). PSS: prestyloid space; RPS: retropharyngeal space; CS: carotid space; PVS: prevertebral space; MMS: medial pterygoid muscle of masticator space; LMS: lateral pterygoid muscle of masticator space and beyond; PS: parotid space.

Based on the above findings, HRs for death according to anatomic site (prestyloid space, carotid space, retropharyngeal space, prevertebral space, medial pterygoid muscle, lateral pterygoid muscle and beyond, and parotid space) were further analyzed to subclassify parapharyngeal extension. There were significant differences in the HR for death between patients with T1 disease (AJCC 2010 staging system) (HR = 1) and those with invasion of other sites. The HRs associated with prestyloid space, carotid space, retropharyngeal space, prevertebral space, and medial pterygoid muscle invasion were similar to each other, whereas those associated with the lateral pterygoid muscle and beyond and parotid space invasion were significantly higher than the others (Figure 2). Therefore, we propose that the parapharyngeal extension should be divided into two grades: mild invasion (involvement of medial pterygoid muscle of masticator space or prestyloid, carotid, prevertebral, or retropharyngeal spaces) and extensive invasion (involvement of lateral pterygoid muscle and beyond of masticator space or parotid space).

### 3.5. Staging Categories for Parapharyngeal Extension

**Figure 3:**
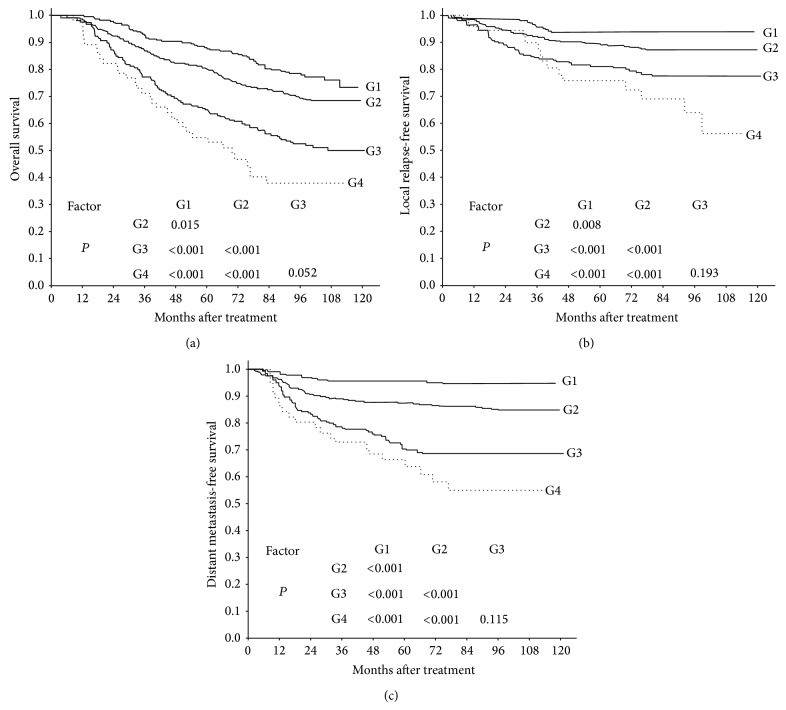
OS (a), LRFS (b), and DMFS (c) curves for patients with NPC stratified according to grade of parapharyngeal extension and T category. G1: mild parapharyngeal extension; G2: T3 without extensive parapharyngeal extension; G3: all T4 (excluding patients staged as T4 because of masticator space invasion); G4: extensive parapharyngeal extension.

To assess the prognostic value and staging categories for different degrees of parapharyngeal extension in NPC, we divided T2–T4 patients (AJCC 2010 staging system) into four groups: group 1, mild parapharyngeal invasion; group 2, T3 without extensive parapharyngeal invasion; group 3, all T4 (excluding patients staged as T4 because of masticator space invasion); and group 4, extensive parapharyngeal extension. No significant difference was observed among these groups with respect to gender, age, N classification, or radiation technique (Table 3). The survival curves for OS, LRFS, and DMFS in these groups are shown in Figure 3. The OS, LRFS, and DMFS of patients with mild parapharyngeal extension were significantly higher than those of T3 patients without extensive parapharyngeal extension (*P* = 0.015, *P* = 0.008, and *P* < 0.001, resp.). By contrast, the OS, LRFS, and DMFS of patients with extensive parapharyngeal extension were close to those of patients with T4 disease (*P* = 0.052, *P* = 0.193, and *P* = 0.115, resp.). Hence, mild and extensive parapharyngeal extension should be classified as stage T2 and T4 disease, respectively.

## 4. Discussion

Parapharyngeal extension of NPC can occur via four pathways: lateral spread to the masticator space or prestyloid space; posterolateral spread to the carotid space or parotid space; posterior spread to the prevertebral space or retropharyngeal space; and inferior spread to the paraoropharyngeal space. In previous studies, investigators have focused on the prognostic significance of parapharyngeal extension in NPC [1–12, 22–24]. However, the results of these studies are somewhat unclear for several reasons. First, the definition of parapharyngeal extension varies and always focuses on a single pathway of spread. For example, the findings reported by Chua et al. [1] and Sham and Choy [2] were based on lateral parapharyngeal spread; by contrast, Ma et al. [3] and Heng et al. [4] proposed that paraoropharyngeal extension should be included in the NPC staging system on the basis of inferior parapharyngeal spread. Second, the outcomes of most studies were based on diagnoses made by CT. With recent advances in the diagnosis of NPC due to the widespread availability of MRI, the management strategy and prognostic factors for this disease have been altered [25–27]. Given these uncertainties, it is important to reassess the prognostic value of parapharyngeal extension and to determine its appropriate subclassification.

To the best of our knowledge, no studies have focused on the subclassification of lateral, posterior, posterolateral, and inferior parapharyngeal spread in NPC. Our study was based on MRI findings and demonstrated that parapharyngeal extension should be subclassified into two risk grades: mild involvement (invasion of the medial pterygoid muscle of the masticator space or the carotid, prestyloid, prevertebral, or retropharyngeal space) and extensive involvement (invasion of the lateral pterygoid muscle of the masticator space and beyond or the parotid space). The OS, LRFS, and DMFS of patients with mild parapharyngeal extension were significantly higher than those of T3 patients, and patients with extensive parapharyngeal extension had OS, LRFS, and DMFS similar to those of patients with stage T4 disease; therefore, it seems reasonable that mild and extensive parapharyngeal extension should be classified as stages T2 and T4 diseases, respectively. This can offer considerable improvement over existing methods for subclassifications of parapharyngeal extension in patients with NPC and has the potential to provide clinicians with reliable information for prognostic prediction and personalizing therapy aimed at NPC patients.

In the fifth and sixth edition AJCC staging system for NPC, staging masticator space involvement was classified as stage T4 disease and defined as lateral tumor extension beyond the anterior surface of the lateral pterygoid muscle [13, 14]. The current, seventh edition includes an adjustment specifying tumor invasion into any part of the anatomic masticator space as stage T4 [15]. Two recent MRI studies [11, 12] showed no significant difference in survival rate between patients with involvement of the medial or lateral pterygoid muscle and patients with involvement of the staging masticator space and suggested that subclassification of masticator space involvement was unnecessary. However, we observed significant differences in OS and DMFS between patients with involvement of the medial pterygoid muscle and patients with involvement of the lateral pterygoid muscle, which has not been reported in previous studies. Therefore, we believe that classification of masticator space spread as involvement of the medial pterygoid muscle versus involvement of the lateral pterygoid muscle and beyond reasonably reflects the prognostic influence of the extent of masticator space spread in NPC.

Carotid space invasion was an important factor in Min's staging system for NPC, with posterolateral tumor invasion beyond a line drawn from the styloid process to the midpoint of the posterior edge of the great occipital foramen classified as stage T3 disease [5]. Two previous studies have reported that the prognostic importance of paraoropharyngeal extension differs from that of paranasopharyngeal extension in NPC [3, 4]. Several researchers have stated that the association of severe carotid space involvement or paraoropharyngeal extension with poor outcomes may be attributed to inadequate dose coverage. When sufficient dose coverage was achieved, carotid space involvement had no significant prognostic value in terms of disease outcome [7–10]. In the present study, all 1504 patients were treated with conventional techniques based on CT simulation, 3DCRT, or IMRT, which ensure better tumor dose coverage. No significant difference in OS, LRFS, or DMFS was observed between patients with anterior carotid space invasion and patients with posterior carotid space invasion or between paraoropharyngeal extension and paranasopharyngeal extension. Therefore, we support the view that subclassification of carotid space involvement or paraoropharyngeal extension in NPC is unnecessary.

The retropharyngeal and prestyloid spaces are close to the nasopharynx and are the most common sites of parapharyngeal extension in NPC. The prevertebral space, located behind the retropharyngeal space, is also a high risk site for tumor involvement. Prevertebral space involvement in hypopharyngeal or laryngeal carcinoma is regarded as unresectable disease (T4b) and is usually associated with poor survival [28]. However, excellent local control and long term survival can be achieved in patients with prevertebral space involvement in NPC if they are treated with curative radiotherapy, especially IMRT. Our data indicate that the OS, LRFS, and DMFS of patients with prevertebral space involvement were approximately similar to those of patients with retropharyngeal or prestyloid space involvement. Therefore, involvement of the prevertebral space, together with the retropharyngeal and prestyloid spaces, may be classified as mild parapharyngeal extension. However, the prognostic importance of parotid space involvement in NPC has not been emphasized in previous studies. Our data indicated that parotid gland involvement was accompanied by lateral tumor spread to the lateral pterygoid muscle, and the prognostic importance of parotid gland involvement in NPC was similar to that of lateral pterygoid muscle invasion.

It should be stressed that pathologic confirmation of imaging findings is not possible in patients with NPC, who are typically treated with radiotherapy rather than surgery, thus determining the anatomic sites of parapharyngeal extension based on MRI alone could be inaccurate. Furthermore, the generality of our results could be reduced by the fact that some patients had incomplete follow-up data and that patients came from an area in southern China with a high incidence of NPC. Therefore, our proposals should be verified in subsequent studies.

## 5. Conclusions

Based on our data, we propose that parapharyngeal extension should be subclassified as mild or extensive and that mild and extensive parapharyngeal extension should be classified as stage T2 and stage T4 disease, respectively. Furthermore, these grades can facilitate staging of NPC and enable a more tailored therapeutic approach with improved outcomes for this disease.

## Figures and Tables

**Table 1:** Characteristics of 1504 patients with nasopharyngeal carcinoma.

Characteristic	Number of patients^*^
WHO pathologic classification	
Type 1	17 (1.1)
Type 2	168 (11.2)
Type 3	1319 (87.7)
T classification	
T1	278 (18.5)
T2	220 (14.6)
T3	587 (39.0)
T4	419 (27.9)
N classification	
N0	344 (22.9)
N1	901 (59.9)
N2	207 (13.8)
N3	53 (3.4)
AJCC 2010 stage	
I	101 (6.7)
II	311 (20.7)
III	638 (42.4)
IVa-b	454 (30.2)

AJCC: American Joint Committee on Cancer; WHO: World Health Organization.

^*^Numbers in parentheses are percentages.

**Table 2:** Incidence of tumor parapharyngeal extension by anatomic site.

Anatomic site	Death		Local failure		Distant metastasis	
	HR^*^ (95% CI)	*P*	HR^*^ (95% CI)	*P*	HR^*^ (95% CI)	*P*
Masticator space						
None	0.551 (0.400–0.760)	<0.001	0.457 (0.267–0.782)	0.004	0.394 (0.256–0.606)	<0.001
Medial pterygoid muscle	0.623 (0.445–0.873)	0.006	0.940 (0.566–1.561)	0.812	0.584 (0.386–0.884)	0.011
Lateral pterygoid muscle	1		1		1	
Staging masticator space	0.906 (0.522–1.573)	0.727	1.147 (0.467–2.819)	0.765	1.067 (0.563–2.023)	0.842
Masticator space						
None	0.885 (0.651–1.202)	0.433	0.486 (0.302–0.780)	0.003	0.675 (0.439–1.037)	0.073
Medial pterygoid muscle	1		1		1	
Lateral pterygoid muscle and beyond	1.572 (1.143–2.163)	0.005	1.091 (0.678–1.754)	0.720	1.734 (1.172–2.567)	0.006
Carotid space						
None	0.735 (0.586–0.921)	0.008	0.693 (0.482–0.996)	0.048	0.689 (0.502–0.947)	0.022
Anterior carotid space	1		1		1	
Posterior carotid space	1.085 (0.787–1.497)	0.618	1.035 (0.802–1.507)	0.621	1.425 (0.952–2.132)	0.085
Parapharyngeal extension						
None	0.678 (0.494–0.931)	0.016	0.659 (0.395–1.099)	0.110	0.832 (0.523–1.325)	0.439
Paranasopharyngeal	1		1		1	
Paraoropharyngeal	1.169 (0.756–1.809)	0.482	1.734 (0.952–3.158)	0.072	1.257 (0.857–2.213)	0.312

^*^HR adjusted for age, gender, chemotherapy, T category, and N category according to the seventh edition AJCC system.

**Table 3:** Characteristics for 1504 patients with different grades of parapharyngeal extension.

Characteristic	Number of patients				*P*
	G1	G2	G3	G4	
Gender					
Male	171	500	202	45	0.203
Female	57	182	51	18	
Age					
<50 y	144	464	163	39	0.419
≥50 y	84	218	90	24	
N classification					
N0	39	153	43	8	0.085
N1	139	414	171	42	
N2-3	50	115	39	13	
Radiation technique					
Conventional technique	120	361	139	31	0.289
IMRT	94	292	104	25	
3DCRT	14	29	10	7	

3DCRT: three-dimensional conformal radiotherapy; IMRT: intensity-modulated radiation therapy; G1: mild parapharyngeal extension; G2: T3 without extensive parapharyngeal extension; G3: all T4 (excluding patients staged as T4 because of masticator space invasion); G4: extensive parapharyngeal extension.

## References

[1] Chua D. T., Sham J. S., Kwong D. L., Choy D. T., Au G. K., Wu P. M. (1996). Prognostic value of parapharyngeal extension of nasopharyngeal carcinoma: a significant factor in local control and distant metastasis. *Cancer*.

[2] Sham J. S. T., Choy D. (1991). Prognostic value of paranasopharyngeal extension of nasopharyngeal carcinoma on local control and short-term survival. *Head and Neck*.

[3] Ma J., Mai H., Hong M. (2001). Is the 1997 AJCC staging system for nasopharyngeal carcinoma prognostically useful for Chinese patient populations?. *International Journal of Radiation Oncology Biology Physics*.

[4] Heng D. M., Wee J., Fong K. W. (1999). Prognostic factors in 677 patients in Singapore with nondisseminated nasopharyngeal carcinoma. *Cancer*.

[5] Huaqing M., Minghuang H., Jun M. (1994). A new staging system for nasopharyngeal carcinoma in China. *International Journal of Radiation Oncology, Biology, Physics*.

[6] Xiao G. L., Gao L., Xu G. Z. (2002). Prognostic influence of parapharyngeal space involvement in nasopharyngeal carcinoma. *International Journal of Radiation Oncology Biology Physics*.

[7] Teo P., Yu P., Lee W. Y. (1996). Significant prognosticators after primary radiotherapy in 903 nondisseminated nasopharyngeal carcinoma evaluated by computer tomography. *International Journal of Radiation Oncology Biology Physics*.

[8] Lee A. W., Foo W., Law S. C. (1999). Staging of nasopharyngeal carcinoma: from Ho's to the new UICC system. *International Journal of Cancer*.

[9] Au J. S. K., Law C. K., Foo W., Lau W. H. (2003). In-depth evaluation of the AJCC/UICC 1997 staging system of nasopharyngeal carcinoma: prognostic homogeneity and proposed refinements. *International Journal of Radiation Oncology Biology Physics*.

[10] Ng W. T., Chan S. H., Lee A. W. M. (2008). Parapharyngeal extension of nasopharyngeal carcinoma: still a significant factor in era of modern radiotherapy?. *International Journal of Radiation Oncology Biology Physics*.

[11] Tang L., Li W., Chen L. (2010). Prognostic value and staging categories of anatomic masticator space involvement in nasopharyngeal carcinoma: a study of 924 cases with MR imaging. *Radiology*.

[12] Sun R., Qiu H., Mai H. (2013). Prognostic value and differences of the sixth and seventh editions of the UICC/AJCC staging systems in nasopharyngeal carcinoma. *Journal of Cancer Research and Clinical Oncology*.

[13] Fleming I. D., Cooper J. S., Henson D. E. (1997). Pharynx (including base of tongue, soft palate, and uvula). *AJCC Cancer Staging Manual*.

[14] Greene F. L., Page D. L., Fleming I. D., Fritz A., Balch C. M. (2002). Pharynx (including base of tongue, soft palate, and uvula). *AJCC Cancer Staging Handbook from the AJCC Cancer Staging Manual*.

[15] Edge S. B., Byrd D. R., Compton C. C., Fritz A. G., Greene F. L., Trotti A. (2010). *AJCC Cancer Staging Handbook from the AJCC Cancer Staging Manual*.

[16] Mao Y., Li W., Chen L. (2009). A clinical verification of the Chinese 2008 staging system for nasopharyngeal carcinoma. *Ai Zheng*.

[17] Zhang L., Zhao C., Peng P. (2005). Phase III study comparing standard radiotherapy with or without weekly oxaliplatin in treatment of locoregionally advanced nasopharyngeal carcinoma: preliminary results. *Journal of Clinical Oncology*.

[18] Zhao C., Han F., Lu L. (2004). Intensity modulated radiotherapy for local-regional advanced nasopharyngeal carcinoma. *Ai Zheng*.

[19] Luo W., Deng X., Lu T. (2004). Dosimetric evaluation for three dimensional conformal, conventional, and traditional radiotherapy plans for patients with early nasopharyngeal carcinoma. *Ai Zheng*.

[20] Kaplan E. L., Meier P. (1958). Nonparametric estimation from incomplete observations. *Journal of the American Statistical Association*.

[21] Cox D. R. (1972). Regression models and life-tables. *Journal of the Royal Statistical Society B: Methodological*.

[22] Feng A., Wu M., Tsai S. Y. C. (2006). Prevertebral muscle involvement in nasopharyngeal carcinoma. *International Journal of Radiation Oncology Biology Physics*.

[23] Lee C.-C., Chu S.-T., Chou P., Chen L.-E. (2008). The prognostic influence of prevertebral space involvement in nasopharyngeal carcinoma. *Clinical Otolaryngology*.

[24] Zhou G., Mao Y., Chen L. (2012). Prognostic value of prevertebral space involvement in nasopharyngeal carcinoma based on intensity-modulated radiotherapy. *International Journal of Radiation Oncology Biology Physics*.

[25] Zhang G., Liu L., Wei W., Deng Y., Li Y., Liu X. W. (2010). Radiologic criteria of retropharyngeal lymph node metastasis in nasopharyngeal carcinoma treated with radiation therapy. *Radiology*.

[26] Liao X., Mao Y., Liu L. (2008). How does magnetic resonance imaging influence staging according to AJCC staging system for nasopharyngeal carcinoma compared with computed tomography?. *International Journal of Radiation Oncology Biology Physics*.

[27] Peng G., Wang T., Yang K. (2012). A prospective, randomized study comparing outcomes and toxicities of intensity-modulated radiotherapy vs. conventional two-dimensional radiotherapy for the treatment of nasopharyngeal carcinoma. *Radiotherapy and Oncology*.

[28] Yousem D. M., Gad K., Tufano R. P. (2006). Resectability issues with head and neck cancer. *American Journal of Neuroradiology*.

